# Pannexin1 channels dominate ATP release in the cochlea ensuring endocochlear potential and auditory receptor potential generation and hearing

**DOI:** 10.1038/srep10762

**Published:** 2015-06-02

**Authors:** Jin Chen, Yan Zhu, Chun Liang, Jing Chen, Hong-Bo Zhao

**Affiliations:** 1 Department of Otolaryngology, University of Kentucky Medical Center, 800 Rose Street, Lexington, Kentucky, 40536, USA; 2 Department of Otolaryngology, Tongji Hospital, Tongji Medical College, Huazhong University of Science and Technology, Wuhan, 430030, P.R. of China

## Abstract

Pannexin1 (Panx1) is a gap junction gene in vertebrates whose proteins mainly function as non-junctional channels on the cell surface. Panx1 channels can release ATP under physiological conditions and play critical roles in many physiological and pathological processes. Here, we report that Panx1 deficiency can reduce ATP release and endocochlear potential (EP) generation in the cochlea inducing hearing loss. Panx1 extensively expresses in the cochlea, including the cochlear lateral wall. We found that deletion of Panx1 in the cochlear lateral wall almost abolished ATP release under physiological conditions. Positive EP is a driving force for current through hair cells to produce auditory receptor potential. EP generation requires ATP. In the Panx1 deficient mice, EP and auditory receptor potential as measured by cochlear microphonics (CM) were significantly reduced. However, no apparent hair cell loss was detected. Moreover, defect of connexin hemichannels by deletion of connexin26 (Cx26) and Cx30, which are predominant connexin isoforms in the cochlea, did not reduce ATP release under physiological conditions. These data demonstrate that Panx1 channels dominate ATP release in the cochlea ensuring EP and auditory receptor potential generation and hearing. Panx1 deficiency can reduce ATP release and EP generation causing hearing loss.

Pannexins and connexins belong to gap junction gene families in vertebrates. However, they have completely different sequences^1^,^2^. Pannexins are homologous to innexins, which encode the gap junction proteins in invertebrates^2^. So far, three pannexin isoforms (Panx1, 2, and 3) have been cloned from the human and mouse genomes^2^. The profile of pannexin channel permeability is similar to that of connexin channels, which allow passage of ions and small molecules up to 1.5 kDa. However, unlike connexins, pannexins usually form non-junctional membrane channels on the cell surface to provide an intracellular-extracellular conduit^3^,^4^,^5^,^6^. Moreover, pannexin channels, particularly Panx1 channels, can open and function at physiological extracellular Ca^++^ levels, at which connexin channels are closed^7^. Panx1 channels can also be activated by mechanical stress, low oxygen, glutamate through NMDA receptors, elevation of extracellular K^+^ concentrations, and ATP binding to purinergic receptors^3^,^8^,^9^,^10^,^11^,^12^,^13^. These specific properties imply that pannexin channels can function in a wider range of physiological conditions.

Pannexins have ubiquitous cellular expression in many tissues and organs, including the inner ear. All three pannexin isoforms are expressed in the mammalian inner ear^14^. Panx1 expresses at the cochlear supporting cells, the spiral limbus, and the cochlear lateral wall. Panx2 only expresses at the basal cell layer in the stria vascularis, and Panx3 expression is restricted to the cochlear bone. These distinct distribution patterns suggest that pannexins play important roles in the inner ear. However, the function of pannexin in the cochlea and hearing has not been characterized yet and remains unclear.

It has been reported that Panx1 channels can release ATP under physiological and pathological conditions^3^,^8^,^10^,^15^,^16^ and play critical roles in many cellular and pathological processes, such as Ca^++^ homeostasis, immunological responses, cell apoptosis, migraine, ischemia, and some neurological disorders^9^,^17^,^18^,^19^,^20^,^21^,^22^,^23^,^24^. ATP also plays important functions in the cochlea and hearing. It has been reported that ATP can elevate intracellular Ca^++^ concentration in hair cells to modify neurotransmission and extend the dynamic range of hearing^25^,^26^,^27^,^28^. We also found that ATP can mediate outer hair cell (OHC) electromotility to regulate hearing sensitivity, gap junctional coupling between the cochlear supporting cells, and K^+^-recycling^29^,^30^,^31^,^32^.

ATP is also required for generation of positive endocochlear potential (EP, +100–110 mV)^33^,^34^,^35^,^36^. Positive EP is generated in the cochlear lateral wall^33^,^36^ and is a driving force that compels K^+^ ions in the endolymph through the transduction channels at stereocilia of hair cells to produce auditory receptor current and potential, thereby initiating hearing. In this study, we found that deletion of Panx1 in the cochlear lateral wall reduced ATP release and EP generation and thereby reduced auditory receptor potential leading to hearing loss. This indicates that Panx1 is required for EP generation and hearing.

## Results

### Panx1 deletion in the cochlea in Panx1 cKO mice

**Figure 1:**
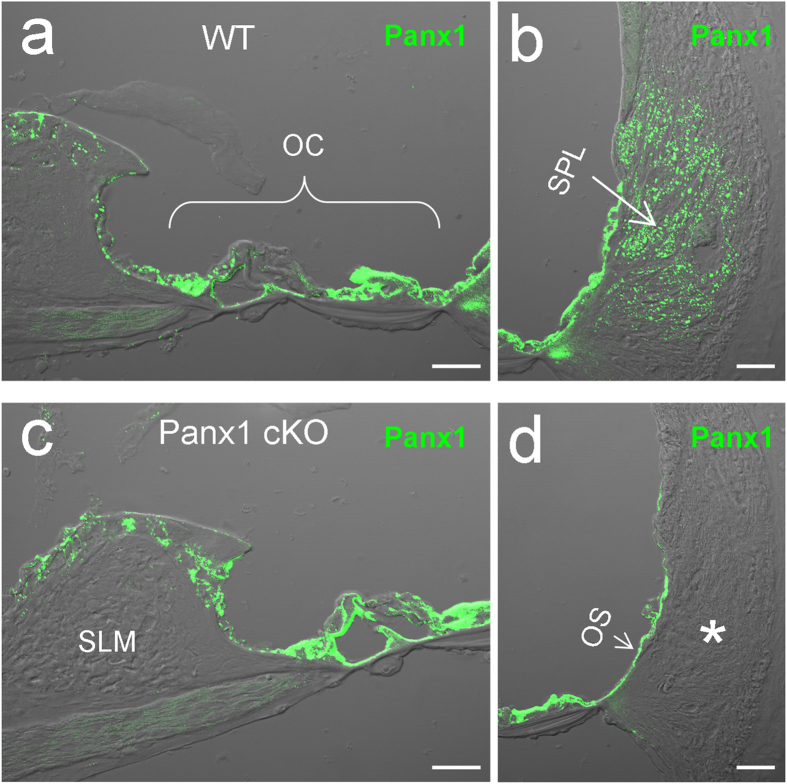
Panx1 deletion in the cochlea in Panx1 conditional knockout (cKO) mice. **a-b**: Immunofluorescent staining for Panx1 in the WT mouse cochlea. **c-d**: Immunofluorescent staining for Panx1 in the Panx1 cKO mouse cochlea. An asterisk indicates that the area of type II fibrocytes in the cochlear lateral wall has no Panx1 labeling. OC: the organ of Corti, OS: outer sulcus cell, SLM: spiral limbus, SPL: spiral ligament. Scale bars: 25 μm.

As we previously reported^14^, Panx1 had extensive expression in the cochlea, including the spiral limbus, the organ of Corti, and the cochlear lateral wall (Fig. 1a,b). In Panx1 conditional knockout (cKO) mice, Panx1 expression at the organ of Corti and the spiral limbus remained. Immunofluorescent staining showed that intense labeling for Panx1 was retained in these regions (Fig. 1c). However, Panx1 labeling at the cochlear lateral wall was absent (Fig. 1d). At the lateral wall of WT mouse cochlea, Panx1 was found to be mainly expressed at the type II fibrocytes in the spiral ligament (SPL) (Fig. 1b). In Panx1 cKO mice, Panx1 labeling at the type II fibrocytes was completely absent (indicated by an asterisk in Fig. 1d).

Deletion of Panx1 did not affect connexin expression. Cx26 and Cx30 are predominant connexin isoforms in the cochlea^37^,^38^,^39^. In Panx1 cKO mice, Cx26 and Cx30 expression at the cochlear lateral wall appeared normal (supplementary Fig. S2). There were no significant differences in Cx26 and Cx30 expression at the cochlear lateral wall between Panx1 cKO mice and WT mice (Cx26: P = 0.38, Cx30: P = 0.13, one-way ANOVA).

### Hearing loss in Panx1 cKO mice

**Figure 2:**
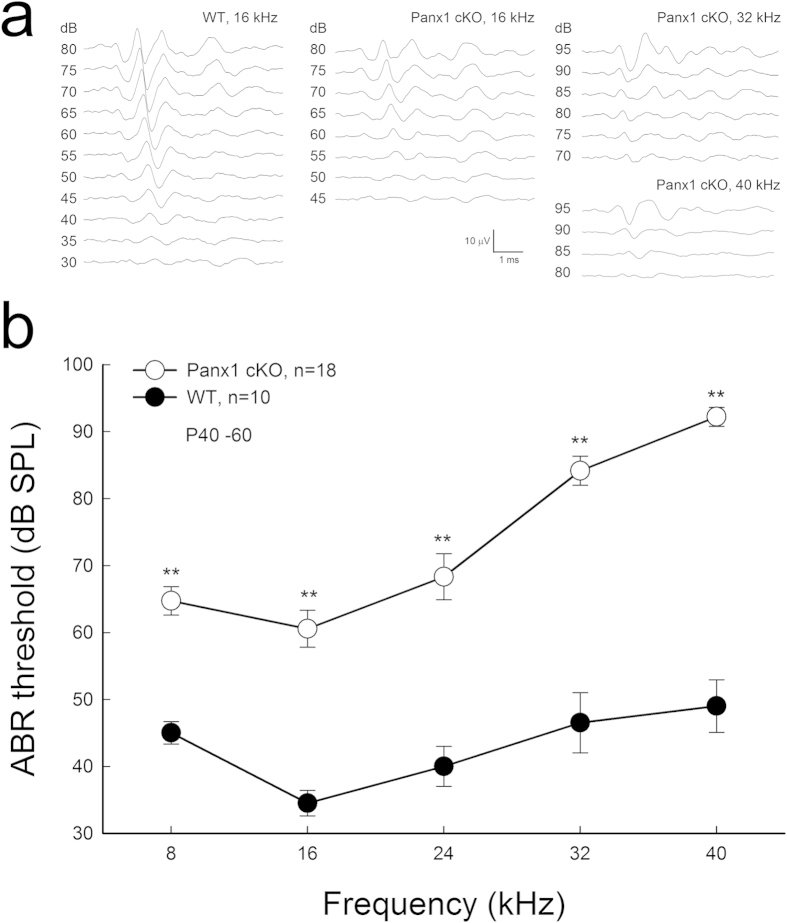
Hearing loss in Panx1 cKO mice. **a**: ABR waveforms recorded from Panx1 cKO and wild-type (WT) mice. **b**: ABR thresholds were measured at age P40-60. ^**^P < 0.001, one-way ANOVA with a Bonferroni correction.

Panx1 cKO mice had hearing loss (Fig. 2). The thresholds of auditory brainstem response (ABR) in Panx1 cKO mice at 8, 16, 24, 32, and 40 kHz were 64.7 ± 2.11, 60.6 ± 2.77, 68.3 ± 3.45, 84.2 ± 2.19, and 92.2 ± 1.41 dB SPL, respectively (Fig. 2b). In comparison with wild-type (WT) mice, the ABR thresholds in Panx1 cKO mice at 8, 16, 24, 32, and 40 kHz were elevated by 19.7 ± 2.11, 26.1 ± 2.77, 28.3 ± 3.45, 37.7 ± 2.19, and 43.2 ± 1.41 dB SPL, respectively (P < 0.001, one-way ANOVA with a Bonferroni correction). The hearing loss appeared severe at higher frequencies.

### EP reduction in Panx1 cKO mice

**Figure 3:**
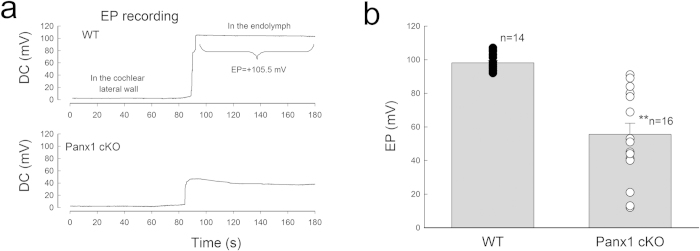
Reduction of EP in Panx1 cKO mice. **a:** Time-sequence of DC potential changes in electrode penetrating through the cochlear lateral wall. **b**: EP reduction in Panx1 cKO mice. EP was recorded at age P40-60. ^**^ P < 0.001, t-test.

The cochlear lateral wall is responsible for generation of positive EP. Fig. 3 shows that deletion of Panx1 at the cochlear lateral wall reduced EP. EP in Panx1 cKO mice and WT mice was 57.6 ± 6.03 and 98.2 ± 1.41 mV, respectively (Fig. 3b). In comparison with WT mice, the EP in Panx1 cKO mice was significantly reduced by 40% (P < 0.001, t-test). However, deletion of Panx1 did not affect the lateral wall development. The thickness of the cochlear lateral wall in Panx1 cKO mice was similar to that in WT mice (P = 0.49, one-way ANOVA, supplementary Fig. S3).

### Reduction of auditory receptor potential in Panx1 cKO mice

**Figure 4:**
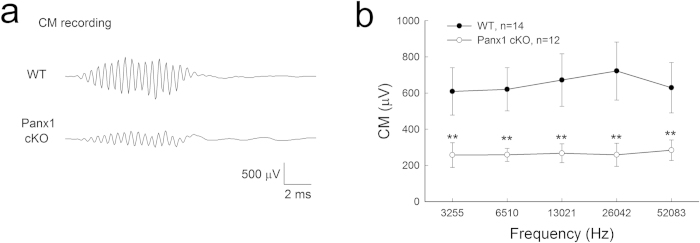
CM reduction in Panx1 cKO mice. Mice were P40-60 old. **a**: The recorded CM waveforms from WT and Panx1 cKO mice. **b**: CM reduction in Panx1 cKO mice. ^**^ P < 0.001, one-way ANOVA with a Bonferroni correction.

EP is the driving force for generation of auditory receptor current and potential. Fig. 4 shows that the auditory receptor potential as measured by cochlear microphonic (CM) in Panx1 cKO mice was reduced. In WT mice, the magnitudes of the recorded CM were 600–700 μV and had a slight increase at 26 kHz near the recording place. In Panx1 cKO mice, the magnitudes of the recorded CM were ~250 μV and significantly reduced by 60–70% (P < 0.001, one-way ANOVA with a Bonferroni correction) as compared with WT mice (Fig. 4b).

### No apparent hair cell degeneration in Panx1 cKO mice

**Figure 5:**
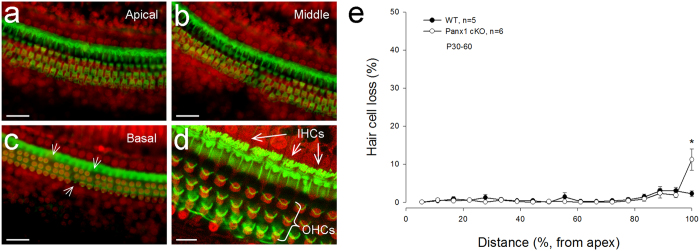
No substantial hair cell degeneration in Panx1 cKO mice. **a-c**: The cochlear sensory epithelia of Panx1 cKO mice staining with phalloidin-Alexa Fluor-488 (green) and propidium iodide (PI, red). White arrows in panel c indicate scattered loss of outer hair cells (OHCs) in the basal turn. Scale bars: 50 μm. **d**: A high-magnification image. Inner hair cell’s (IHC) and OHC’s hair bundles are clearly visible. Scale bars: 20 μm. **e**: Hair cell loss accounting in Panx1 cKO and WT mice. Mice were P60-90 old. ^*^ P < 0.05, one-way ANOVA with a Bonferroni correction.

Fig. 5 shows that there was no apparent hair cell loss in Panx1 cKO mice. At the end of the basal turn (very high frequency region), Panx1 cKO mice had a significant increase in hair cell loss as compared with WT mice (P = 0.022, one-way ANOVA with a Bonferroni correction). However, the loss of hair cells was less than 10% (Fig. 5e), and could not produce broad, severe hearing loss as shown in Fig. 2.

### Reduction of ATP release in Panx1 cKO mice

**Figure 6:**
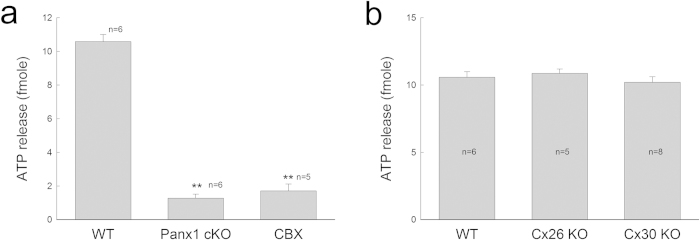
Reduction of ATP release in the cochlea in Panx1 cKO mice. Mice were P40-60 old. **a:** ATP release is reduced in Panx1 cKO mice and inhibited by application of 0.1 mM CBX. ^**^P < 0.001, one-way ANOVA with a Bonferroni correction. **b:** Connexin deletion does not reduce ATP release in the cochlea.

EP generation requires ATP^34^,^35^. Fig. 6 shows that deletion of Panx1 at the cochlear lateral wall almost abolished ATP release under physiological conditions. At 2 mM extracellular Ca^++^ concentration, the amount of ATP release in Panx1 cKO and WT mice was 1.28 ± 0.23 and 10.58 ± 0.42 fmoles, respectively (Fig. 6a). Compared to WT mice, ATP release in Panx1 cKO mice was significantly reduced by ~8-fold (P < 0.001, one-way ANOVA with a Bonferroni correction). Moreover, application of carbenoxolone (CBX), which can block pannexin channels^7^, could also reduce ATP release in WT mice (Fig. 6a). The amount of ATP release under 0.1 mM CBX treatment in the WT mice was significantly reduced to 1.71 ± 0.41 fmoles (P < 0.001, one-way ANOVA with a Bonferroni correction), similar to values obtained in Panx1 cKO mice. However, deletion of Cx26 or Cx30 did not reduce ATP release under the physiological level of Ca^++^ concentration (Fig. 6b). The measured ATP release at 2 mM extracellular Ca^++^ concentration in Cx26 KO mice and Cx30 KO mice was 10.87 ± 0.32 and 10.21 ± 0.41 fmoles, respectively. There was no significant difference in the amount of ATP released from WT and Cx26 KO mice or Cx30 KO mice (P = 0.98, one-way ANOVA).

## Discussion

In this study, we found that Panx1 deficiency can cause hearing loss (Figs. 1 and 2). Deletion of Panx1 at the cochlear lateral wall abolished ATP release, reduced EP and auditory receptor potential generation, and eventually led to hearing loss (Figs. 3,4,6). These data reveal that Panx1 channel-mediated ATP release is required for EP generation and auditory receptor potential producing. This study also provides the first evidence that Panx1 is required for hearing.

**Figure 7:**
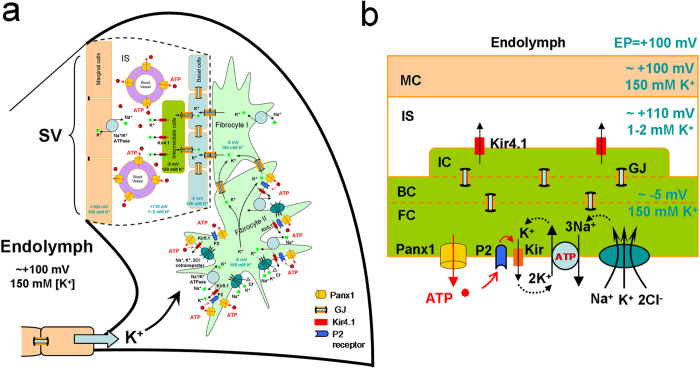
Schematic drawing of EP generation with Panx1 ATP release in the cochlear lateral wall. **a:** Based on the “two-cell” model, EP is generated by Kir4.1 in the apical membrane of intermediate cells in conjunction with Kir5.1 channels, Na^+^/K^+^ ATPases, and Na^+^, K^+^, 2Cl^-^-cotransporters in the fibrocytes through gap junctional coupling. **b:** A simplified “two-cell” model of EP generation with Panx1 ATP release. ATP may activate purinergic (P2) receptors and then subsequently activate Kir K channels to ensure Na^+^/K^+^-ATPase activity and Na^+^, K^+^, 2Cl^-^ cotransporter activity to produce EP. MC: marginal cell; BC: basal cell; FC: fibrocyte; IC: intermediate cell; IS: intrastrial space; GJ: gap junction.

Positive EP (+100-110 mV) in the cochlear endolymph is a driving force that propels K^+^ ions through transduction channels in hair cells to produce auditory receptor current and potential. Positive EP is generated in the cochlear lateral wall by a complex process^33^,^36^. Based on a widely accepted “two-cell” model (Fig. 7), EP generation is initiated at fibrocyte cells in the spiral ligament. Na^+^/K^+^-ATPases and Na^+^, K^+^, 2Cl^-^ cotransporters at the type II fibrocyte cells depolarize cells to ~ –5 mV. Then, the intermediate cells in the stria vascularis (SV) are consequently depolarized to ~ –5 mV through gap junctional coupling, which is formed by Cx26 and Cx30^39^. Subsequently, ATP-dependent Kir4.1 K^+^ channels at the apical membrane of the intermediate cells 39,40,41 generate a 105-110 mV transmembrane potential (Nernst’s K^+^ equilibrium potential) between the intracellular space and the intrastrial space, i.e., +110-115 mV in the intrastrial space with respect to normal extracellular space, since the K^+^ concentration in the intrastrial space is lower (1-2 mM). Finally, this positive intrastrial potential eventually leads to positive EP (+100-110 mV) in the endolymph in the scala media (Fig. 7).

Evidently, ATP is necessary and required for EP generation. It has been reported that intracochlear perfusion of ATP can significantly increase EP^42^. As mentioned above, depolarization of fibrocyte cells by co-activation of Na^+^/K^+^-ATPases and Na^+^, K^+^, 2Cl^–^ -cotransporters is the first step for EP generation. Although Na^+^/K^+^-ATPase is driven by intracellular ATP, it has been reported that extracellular ATP can also stimulate Na^+^/K^+^-ATPase activity through activation of purinergic receptors and Src family kinase (SFK)^43^. Moreover, the function of the primary active Na^+^/K^+^-ATPase requires K channel coupling to recycle K^+^ (Fig. 7b). This “pump coupling” was postulated in 1958 and later corroborated by experimental data^44^,^45^,^46^. Recently, it has been found that extracellular ATP can also activate ATP-sensitive (Kir) K channels in hippocampal CA3 pyramidal neurons and lung epithelial cells to co-operate with Na^+^/K^+^-ATPases activity^47^,^48^. ATP may primarily activate P2X receptors and then subsequently activate Kir K channels and Na^+^/K^+^-ATPases (Fig. 7b). In this study, we found that Panx1 deletion reduced ATP release and EP (Figs. 3and 6). These findings indicate that Panx1 channel-mediated ATP release plays an important role in the EP generation. We previously also found that ATP is required for K^+^-recycling in the cochlea^31^,^32^. Deletion of Panx1 reduced ATP release (Fig. 6) and could also consequently compromise K^+^-recycling to impair EP generation (Fig. 7a).

In this study, we found that deletion of Panx1 or application CBX in WT mice abolished ATP release in the cochlear lateral wall at physiological extracellular Ca^++^ (2 mM) levels (Fig. 6). CBX can block both connexin and pannexin channels^7^,^13^. However, the connexin hemichannels are already closed at this high Ca^++^ level^7^. Thus, CBX in this experiment mainly blocked Panx1 channel activity. These data indicate that Panx1 channels dominate ATP release in the cochlear lateral wall under physiological conditions, even thought other ATP release mechanisms may exist. This concept is further supported by the fact that Cx26 or Cx30 deletion did not reduce ATP release at 2 mM physiological extracellular Ca^++^ level (Fig. 6b), at which concentration connexin hemichannels are closed^7^. Thus, deletion of Cx26 or Cx30 had little effect on ATP release under physiological conditions (Fig. 6b). Cx26 or Cx30 deletion could also reduce or abolish EP. However, the EP reduction results from the disruption of gap junctional coupling^49^,^50^.

Gap junctions play an important role in hearing. Connexin mutations can induce a high incidence of hearing loss, responsible for >50% of nonsyndromic deafness^51^. However, pannexin mutation-induced hearing loss in humans has not been identified yet. In this study, we found that Panx1 deficiency could cause hearing loss in mice (Fig. 2). This strongly suggests that Panx1 deficiency may be able to induce hearing loss in humans as well, which requires further study in the future.

## Materials and Methods

### Creation of Panx1 cKO mice and genotyping

Panx1^tm1a(KOMP)Wtsi^ mice, in which Exon2 is floxed with loxPs (supplementary Fig. S1a), were purchased from KOMP (Knock Out Mouse Project, David, CA). They have a hypomorphic phenotype^52^ and retain Panx1 expression in the cochlea (supplementary Fig. S4). We further crossed Panx1^tm1a(KOMP)Wtsi^ mice with Foxg1-Cre transgenic mice (Stock No. 004337, Jackson Lab, http://jaxmice.jax.org/strain/004337.html) to create Panx1 cKO mice. The genotyping of Panx1^tm1a(KOMP)Wtsi^ mice was identified by PCR amplification with the following primers: Panx1-Mut1 (common mutant primer): 5’-GAG ATG GCG CAA CGC AAT TAA T-3’, Panx1-Mut2 (gene specific primer): 5’-CTG GCT CTC ATA ATT CTT GCC CTG-3’, Panx1-WF (wildtype-F): 5’-CTG TAT CAC ACA ACC ACT TCA GAG AAG G-3’, and Panx1-WR (wildtype-R): 5’-GAG CTG ACC CCT TTC CAT TCA ATA G-3’, and generated a 579 bp WT band and a 381 bp mutation band (supplementary Fig. S1b). The Cre transgene in the generated Panx1-Foxg1 mice was identified using following primers: CreF: 5’-GCA TTA CCG GTC GAT GCA-3’ and CreR: 5’-GAA CCT GGTCGA AAT CAG-3’, which produced ~400 bp positive band for the *Cre* transgene (Fig. S1c). The post-Cre Exon2-deleted Panx1 allele in Panx1-Foxg1 mice was identified by Panx1-Mut1a (post-Cre mutant primer): 5’-CAC TGC ATT CTA GTT GTG GTT TGT CC-3’ and Panx1-Mut2 (gene specific primer): 5’-CTG GCT CTC ATA ATT CTT GCC CTG-3’, which generated a 421 bp band after deletion of Exon2. The reaction would be negative for WT or no Exon2 deletion (supplementary Fig. S1d). We used homozygous *Foxg1*^*Cre*^*:Panx1*^*f/f*^ mice as Panx1 cKO mice in this study. The WT littermate mice served as WT controls in the experiment. The experimental procedures were approved by the University of Kentucky’s Animal Care & Use Committee and conducted according to the standards of the NIH Guidelines for the Care and Use of Laboratory Animals.

### Cx26 KO and Cx30 KO mice

As we previously reported^53^, Cx26 KO mice were generated by crossing Cx26^*loxP/loxP*^ mice (EM00245, European Mouse Mutant Archive) with the Pax2-Cre mouse line (the Mutation Mouse Regional Center, Chapel Hill, NC). The Cx26 floxed allele and Pax2-Cre transgene were detected on tail genomic DNA by PCR amplification using the following primers: Cx26F: 5’-CTT TCC AAT GCT GGT GGA GTG-3’ and Cx26R: 5’-ACA GAA ATG TGT TGG TGA TGG-3’ for the Cx26 floxed allele; Pax2-CreF: 5’-GCC TGC ATT ACC GGT CGA TGC AAC GA- 3’ and Pax2-CreR: 5’-GTG GCA GAT GGC GCG GCA ACA CCA TT- 3’ for Pax2-Cre transgene. *Cx26*^*loxP/loxP*^ and WT mice generated 400 and 300 bps bands, respectively. The band of Pax2-Cre was 700 bps.

Cx30 KO mice were also purchased from European Mouse Mutant Archive (EM00323^49^,^50^, ). Primer pairs for detecting Cx30 KO were Cx30 KO-1 (LACZ e Neo): 5’-GGT ACC TTC TAC TAA TTA GCT TGG -3’; Cx30 KO2 (LACZ e Neo): 5’-AGG TGG TAC CCA TTG TAG AGG AAG -3’; Cx30 KO-3 (LACZ e Neo) 5’-AGC GAG TAA CAA CCC GTC GGA TTC -3’. The bands of Cx30 KO and WT mice are located at 460 and 544 bps, respectively.

### ABR measurement

ABR was measured by a Tucker-Davis’ ABR workstation (Tucker-Davis Tech. Alachua, FL)^50^,^53^,^54^,^55^. Mice were anaesthetized by intraperitoneal injection with a mixture of ketamine and xylazine (8.5 ml saline + 1 ml Ketamine + 0.55 ml Xylazine, 0.1 ml per 10 g). Body temperature was maintained at 37–38 ^o^C. Two subdermal needle electrodes were inserted at the vertex (an active electrode) and ventrolaterally to the right or left ear (a reference electrode). The ground needle electrode was inserted in the right leg. ABR was measured by clicks in alternative polarity and series tone bursts (8 – 40 kHz) from 80 to 10 dB SPL in a 5-dB step. The ABR threshold was determined by the lowest level at which an ABR can be recognized. If the ABR threshold was greater than 75 dB SPL, the acoustic stimuli from 110 to 70 dB SPL were added.

### EP and CM recording

Mice were anaesthetized as described above and the body temperature was maintained at 37–38 ^o^C. The trachea was exposed and cut along the middle line. The tracheal tube was put into the trachea. Then, the cochlea was exposed by a ventral approach and the bone over the spiral ligament was gently picked to form a small hole^50^,^54^,^55^. A glass pipette filled with a K^+^-based intracellular solution was inserted into the hole. The DC potential was continually recorded as the electrode pipette penetrated through the lateral wall (Fig. 3a).

For CM recording, acoustic tone bursts (3.25 – 52 kHz) were delivered through an ES-1 high frequency speaker (Tucker Davis Tech. FL). The CM response was recorded by MultiClamp 700A amplifier (Molecular Devices, CA) and digitized utilizing a Digidata 1322A (Molecular Devices, CA).

### ATP release measurement

As we previously reported^29^, the mouse temporal bone was micro-dissected in a sterile normal extracellular solution (ECS) (142 NaCl, 5.37 KCl, 1.47 MgCl_2_, 2 CaCl_2_, 10 HEPES in mM, osmolarity 310 mOsm and pH 7.2). The inner ear was opened from its apex to base. After removal of the bone, the exposed cochlear lateral wall was dissected and put into an incubation chamber. For testing Panx1 channel ATP release, the isolated lateral wall was incubated in 200 μL ECS, which contains 2 mM Ca^++^ and 1.47 mM Mg^++^ to block connexin hemichannels, for 10 minutes. Every 5 minutes, the whole incubation solution (200 μL) was collected and replaced with fresh solution. The osmolarity of all solutions was measured by a micro-computer controlled osmometer (Model 3300, Advanced Instruments Inc. Norwood, MA) and adjusted by Dextrose. All experiments were performed at room temperature (23 ^o^C).

The collected incubation solutions were kept on ice. As we previously reported^29^, the amount of ATP was measured by a bioluminescence method with a luciferin-luciferase assay kit (FL-ASC, Sigma) using a black 96-well plate to avoid optical cross-talk. The bioluminescence was read by a Biotek Synergy 4 Hybrid Microplate Reader (Biotek Instruments Inc, Winooski, VT, USA). The amount of ATP was calculated from the ATP standard curve, which was established from measurements of serial-diluted ATP standards (supplementary Fig. S5). The standard curve also served as an internal control for bioluminescence measurement.

### Immunofluorescent staining and quantitative measurement

The detailed methods and procedures of immunofluorescent staining can be found in our previous reports^14^,^38^,^39^. The cochlea was fixed with 4% paraformaldehyde. The cochlear cryostat sections were washed with PBS for 5 min twice and incubated in a blocking solution (10% goat serum and 1% bovine serum albumin) with 0.1% Triton X-100 for 30 min. Then, the tissue sections were incubated with chicken anti-human Panx1 antibody (1:500; #4515, a gift from Dr. Gerhard Dahl at the University of Miami Medical School) in the blocking solution overnight. For double immunofluorescent staining, monoclonal mouse anti-Cx26 antibody (1: 400, Cat# 33-5800, Invitrogen, CA) and polyclonal rabbit anti-Cx30 antibody (1:400, #71-2200, Invitrogen, CA) were used. After being washed with PBS three times, the sections were reacted with corresponding Alexa Fluor 488- or 568 secondary antibodies (1:500, Molecular Probes) for 2 hr at room temperature (23 ^o^C). The staining was observed under a fluorescent microscope or laser confocal microscope. The images were saved in TIFF format for analysis and presentation.

For quantitative measurement of Cx26 and C30 labeling at the cochlear lateral wall, ImageJ software (NIH, Bethesda, MD) was used^38^,^55^. The density of labeling at the lateral wall was measured and compared between WT and Panx1 cKO mice.

### Statistical analysis

Data were expressed as mean ± s.e.m. unless otherwise indicated in text and plotted by SigmaPlot (SPSS Inc. Chicago, IL). The statistical analyses were performed by SPSS v18.0 (SPSS Inc. Chicago, IL) using one-way ANOVA with a Bonferroni correction or t-test.

## Additional Information

**How to cite this article**: Chen, J. *et al.* Pannexin1 channels dominate ATP release in the cochlea ensuring endocochlear potential and auditory receptor potential generation and hearing. *Sci. Rep.* **5**, 10762; doi: 10.1038/srep10762 (2015).

## Supplementary Material

Supplementary Information
