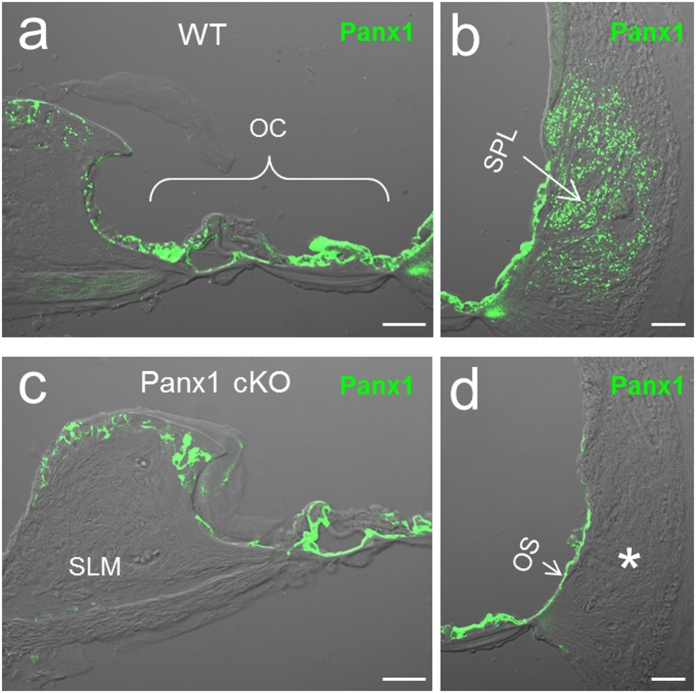# Correction: Corrigendum: Pannexin1 channels dominate ATP release in the cochlea ensuring endocochlear potential and auditory receptor potential generation and hearing

**DOI:** 10.1038/srep46898

**Published:** 2017-08-24

**Authors:** Jin Chen, Yan Zhu, Chun Liang, Jing Chen, Hong-Bo Zhao

**Keywords:** Cellular neuroscience, Cell biology

10.1038/srep10762

This Article contains an error in Figure 1, where panel 1C was duplicated from Figure 1A in Reference 1.

The correct Figure 1 appears below. All the other panels remain unchanged. The conclusions of the Article are unaffected by the correction of panel C.

**Figure 1:**