# Supplementary material for: Pannexin1 channels dominate ATP release in the cochlea ensuring endocochlear potential and auditory receptor potential generation and hearing
Source: Sci Rep. 2015 Jun 2;5:10762. doi: 10.1038/srep10762 (PMC4451810; doi:10.1038/srep10762)
Supplement: Supplementary Information [file srep10762-s1.pdf]

Supplementary figures:

Pannexin1 channels dominate ATP release in the cochlea ensuring endocochlear potential and auditory receptor potential generation and hearing

Jin Chen[1,2]*, Yan Zhu[1]*, Chun Liang[1]*, Jing Chen[1], Hong-Bo Zhao[1]

[1]Department of Otolaryngology, University of Kentucky Medical Center, 800 Rose Street, Lexington, Kentucky, USA, 40536

[2]Department of Otolaryngology, Tongji Hospital, Tongji Medical College, Huazhong University of Science and Technology, Wuhan, P.R. of China, 430030

* Equal contributors

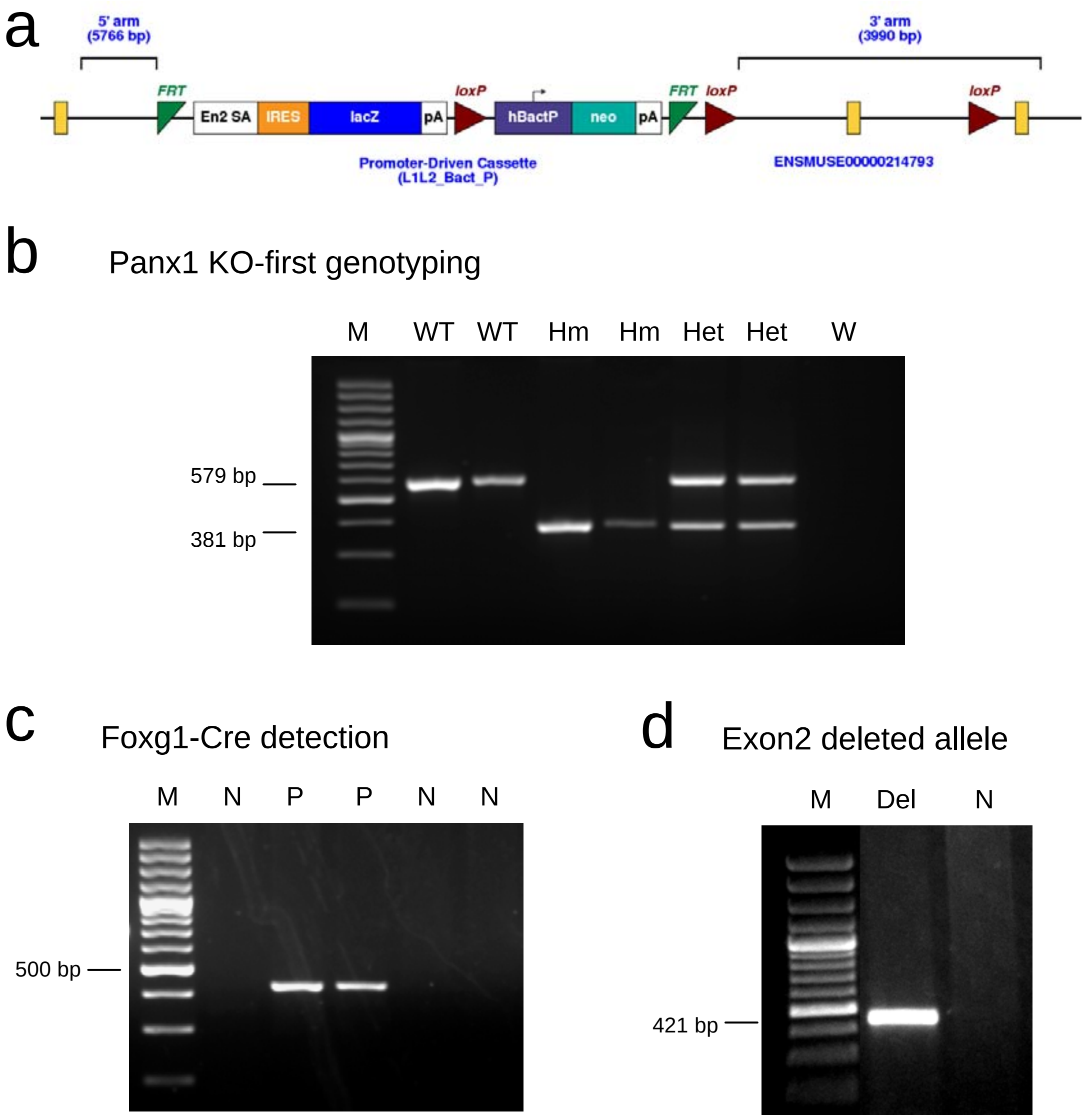

Fig. S1. Knockout Strategy and genotyping of Panx1 KO-first and cKO mice.

a: Structure of KOMP Panx1 KO-first mice. Exon2 is floxed with loxPs.

b: Genotyping of Panx1 KO-first mice. M: marker; Hm: homozygous; Het: heterozygous; W: water.

c: Foxg1-Cre genotyping. N: negative; P: positive.

d: Detection of post-Cre Exon2 deleted Panx1 allele. Mutant reaction after deletion of floxed Exon2 produces a 421 bp band and would be negative at WT or if the Exon2 is not deleted. Del: deleted. N: negative.

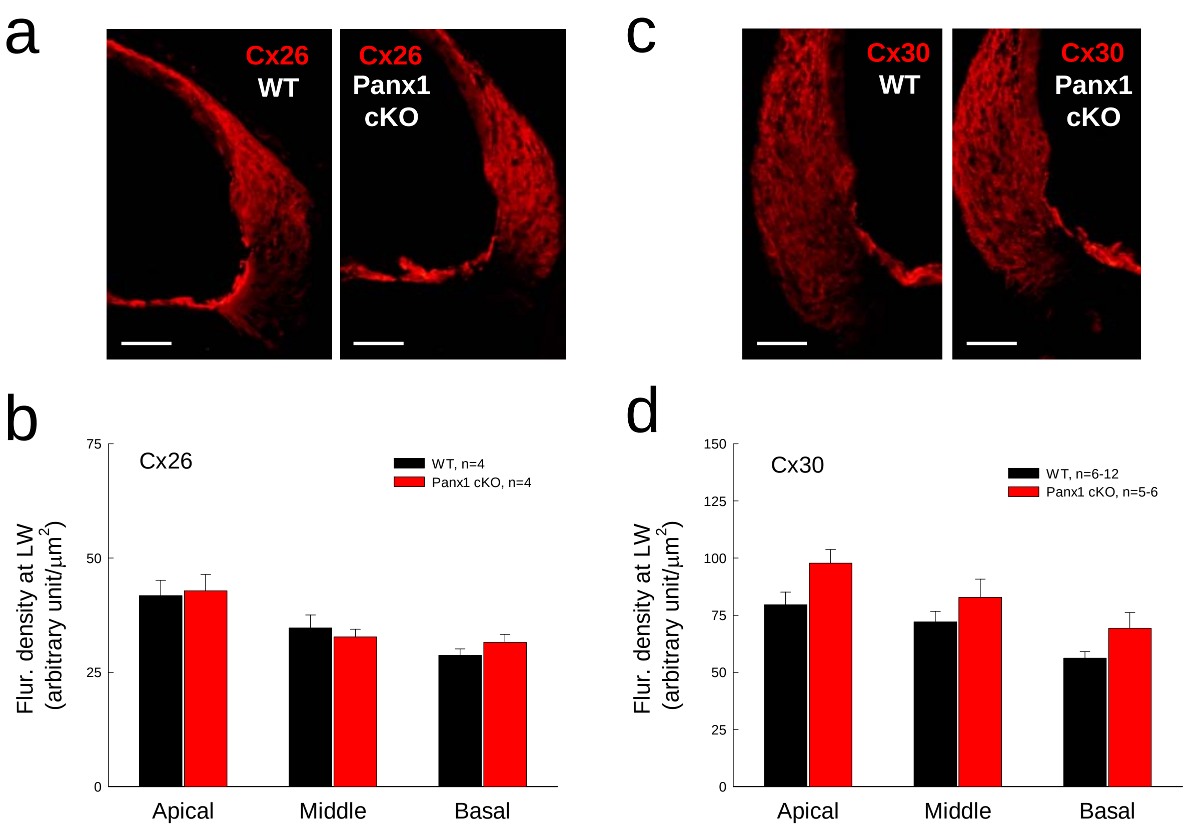

Fig. S2. Normal expression of Cx26 and Cx30 at the cochlear lateral wall in Panx1 cKO mice.

a,c: Immunofluorescent staining for Cx26 and Cx30 at the cochlear lateral wall in the middle turn. Scale bar: 200 μm.

b,d: Quantitative measurement of Cx26 and Cx30 expression at the lateral wall. In comparison with WT mice, Cx26 and Cx30 expression at the lateral wall in Panx1 cKO mice has no significant difference (Cx26: $P=0.38$, Cx30: $P = 0.13$, determined by one-way ANOVA).

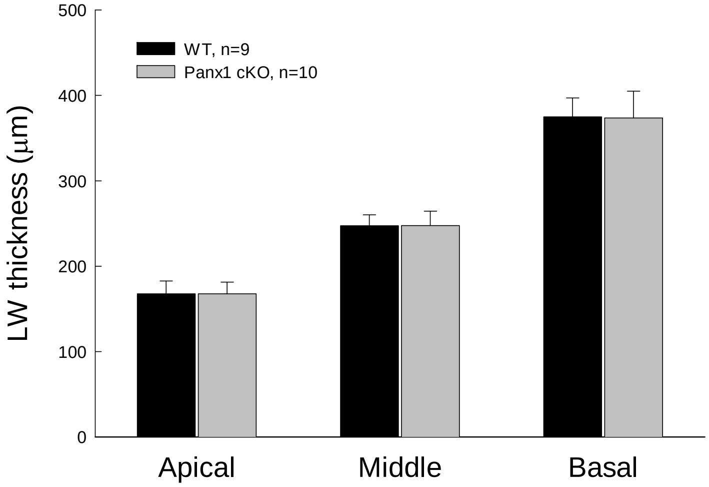

Fig. S3. The lateral wall (LW) thickness measured in WT and Panx1 cKO mice. Mice were P40-80 old. The thickness of the lateral wall in Panx1 cKO mice is not reduced.

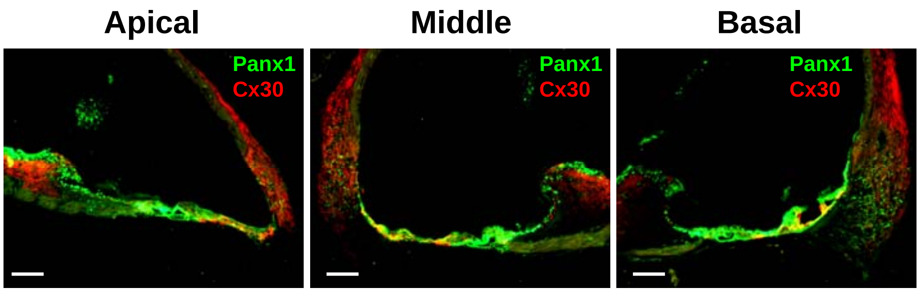

Fig. S4. Immunofluorescent staining for Panx1 in Panx1$^{tm1a(KOMP)Wtsi}$ mice. Intense labeling for Panx1 is visible in the cochlea. Scale bar: 200 µm.

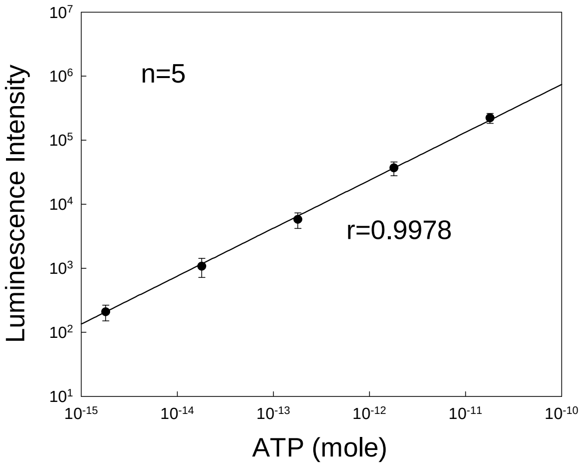

Fig. S5. Standard curve of ATP amount measurement with bioluminescence assay. A solid line represents linear fitting: $y=7.41x10^{15} * x + 139.29$.
